# The origins of Atlantic salmon (*Salmo salar* L.) recolonizing the River Mersey in northwest England

**DOI:** 10.1002/ece3.353

**Published:** 2012-09-11

**Authors:** Charles Ikediashi, Sam Billington, Jamie R Stevens

**Affiliations:** 1 School of Biosciences, University of ExeterGeoffrey Pope Building, Stocker Road, Exeter, EX4 4QD, UK; 2 Environment Agency (North West Region)Richard Fairclough House, Knutsford Road, Warrington, WA4 1HG, UK

**Keywords:** Genetic assignment, Irish Sea, Mersey, microsatellite, straying, Salmo salar

## Abstract

By the 1950s, pollution had extirpated Atlantic salmon in the river Mersey in northwest England. During the 1970s, an extensive restoration program began and in 2001, an adult salmon was caught ascending the river. Subsequently, a fish trap was installed and additional adults are now routinely sampled. In this study, we have genotyped 138 adults and one juvenile salmon at 14 microsatellite loci from across this time period (2001–2011). We have used assignment analysis with a recently compiled pan-European microsatellite baseline to identify their most probable region of origin. Fish entering the Mersey appear to originate from multiple sources, with the greatest proportion (45–60%, dependent on methodology) assigning to rivers in the geographical region just north of the Mersey, which includes Northwest England and the Solway Firth. Substantial numbers also appear to originate from rivers in western Scotland, and from rivers in Wales and Southwest England; nonetheless, the number of fish originating from proximal rivers to the west of the Mersey was lower than expected. Our results suggest that the majority of salmon sampled in the Mersey are straying in a southerly direction, in accordance with the predominantly clockwise gyre present in the eastern Irish Sea. Our findings highlight the complementary roles of improving water quality and in-river navigability in restoring salmon to a river and underlines further the potential benefits of restoration over stocking as a long-term solution to declining fish stocks.

## Introduction

**Figure 1:**
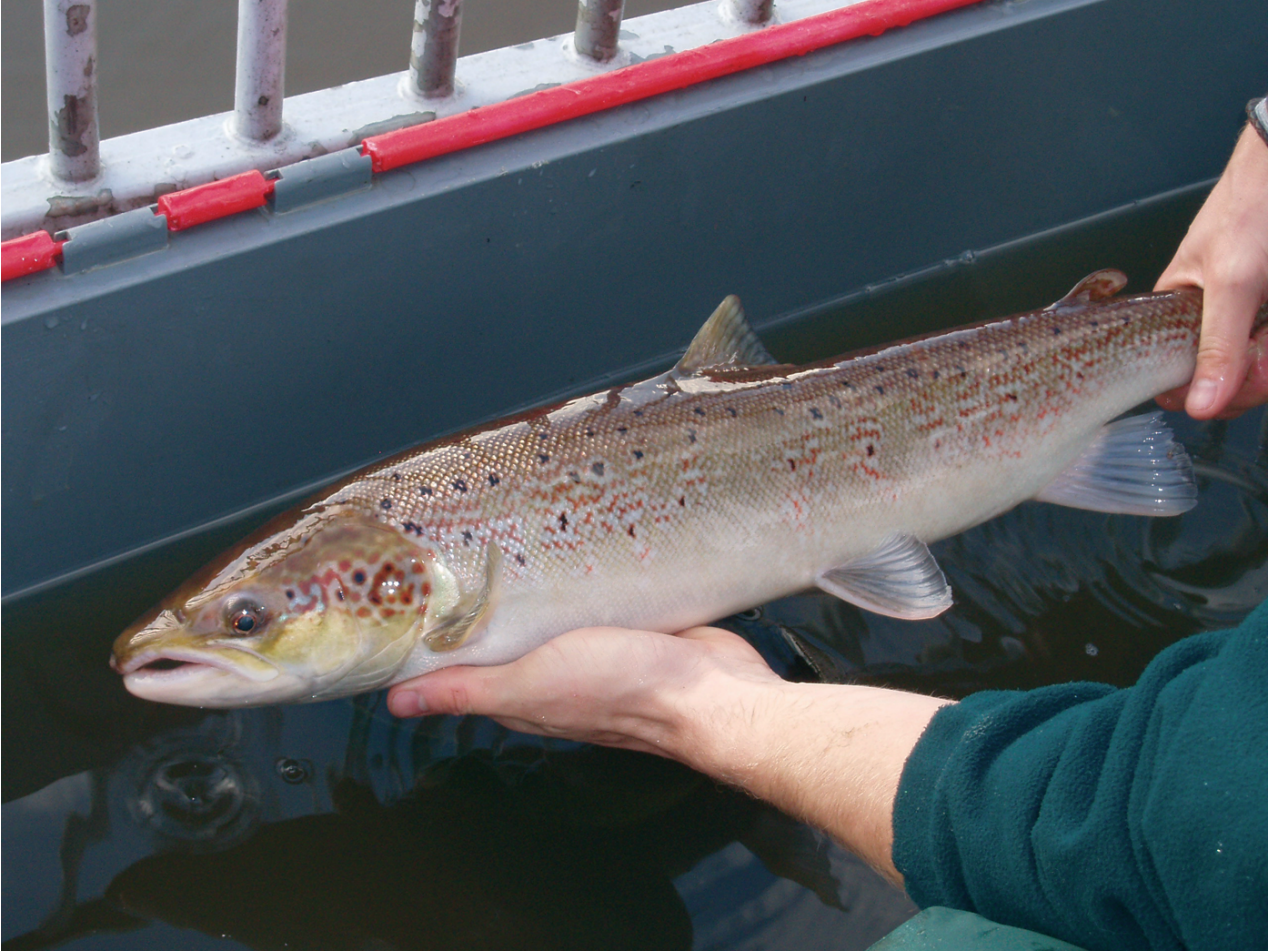
Sampling adult Atlantic salmon from the river Mersey.

Global catch data show that Atlantic salmon, *Salmo salar* L. (Fig. 1), like many other fish have been in steep decline since the 1970s (e.g., Parrish et al. 1998). The reasons appear to be multi-factorial, but include pollution and related reductions in water quality (Thorstad et al. 2007), reduction in access to waterways in which salmon spawn (e.g., Ugedal et al. 2008; Lin 2011), and an uncertain degree of marine mortality (Friedland 1998; Friedland et al. 2000).

Largely because of their iconic status and commercial value, huge amounts of money have been spent on reversing this downward trend, and a large proportion of this funding has been channeled through the controversial measure of stocking with hatchery-bred fish (Milner et al. 2004; Fraser 2008). Despite a clear lack of evidence regarding the success of stocking practices (e.g., Finnegan and Stevens 2008; Fraser 2008; McGinnity et al. 2009), it continues to be seen as a rapid solution to declining fish numbers by a significant number of fishery managers. Yet, in the light of genetic advances, stocking has come under further scrutiny as the limitations and, in many cases, negative impacts of the practice on the genetic diversity and population structure of endemic populations are revealed (Ayllon et al. 2006; Hutchings and Fraser 2008; Griffiths et al. 2009).

At the same time, the value of river restoration (in terms of both improved water quality and river access) is being recognized as a viable alternative, which can subsequently facilitate natural recolonization. Examples have been reported for trout (*Salmo trutta*) in Norway (Knutsen et al. 2001) and Germany (Schreiber and Diefenbach 2005), Atlantic salmon (*Salmo salar*) in France (Perrier et al. 2010), and coho salmon (*Oncorhynchuskisutch*) in Pacific northwestern USA (Anderson and Quinn 2007; Kiffney et al. 2009). Most recently, a study by Griffiths et al. (2011), using microsatellite analysis, demonstrated no trace of the hundreds of thousands of Scottish and Irish-origin hatchery salmon stocked into the river Thames since 1975 (Griffiths et al. 2011); instead, all of the salmon caught in the Thames since 2003, were identified (by assignment analysis) as having originated in other proximal rivers in southern England. Genetic assignment methods have also been used to identify the origins of Atlantic salmon in the river Selja in Estonia (Vasemägi et al. 2001), the river Tambre in Spain (Saura et al. 2008), and the river Seine in France (Perrier et al. 2010), all of which have shown recent returns of Atlantic salmon after years of absence. In the cases of the Seine and the Thames, salmon appear to have returned naturally via straying after attempts at restocking were unsuccessful. If restoration is to be considered as a viable alternative to restocking for restoring Atlantic salmon, then more documented cases of natural recolonization are required. In this regard, the river Mersey in northwest England presents an excellent case study.

The Mersey, which passes through the major urban areas of Liverpool and Manchester, suffered greatly as a result of the Industrial Revolution (see Jones 2000, 2006 for full review). The 1820s saw the expansion of several industries (Gregory et al. 1953; Burton 2003; Lawton and Smith, 1953) and industrial prosperity attracted huge numbers of people to the area (Jones 2006). Subsequent pollution had serious effects on fish stocks and by the 1850s, fish were reportedly absent from the river Irwell, a major tributary of the river Mersey (Bracegirdle 1973; Holland and Harding 1984). Growth continued until the 1960s, particularly around the Mersey estuary and anecdotal evidence suggests that by the 1950s, there were no fish in the river (Wilson et al. 1988 and Jones 2000).

Water quality only began to improve in the 1970s, when a range of new legislation related to water quality was introduced. In 1983, a conference focusing on the Mersey was convened, which led to the creation of the Mersey basin campaign (Jones 2000, 2006; Burton 2003). This heralded many changes that led to the Mersey becoming one of Britain's most high profile environmental success stories, earning the inaugural International Thiess River prize in 1999 for best river system clean-up.

Although there is anecdotal evidence that salmonids began entering the Mersey estuary as early as the 1980s (Wilson et al. 1988), it is likely that salmon began entering the river Mersey in the early 1990s as a result of the improving water quality (Jones 2000; Burton 2003). Video evidence of salmonids attempting to negotiate weirs on the river Bollin, a tributary of the Manchester Ship Canal, was taken in 1999 and 2000 (Jones 2006; Environment Agency, unpublished data), and in 2001, the first salmon in several decades was caught by the Environment Agency (Jones 2006). Critically, no stocking or translocations of salmon are recorded from this period (Environment Agency 2000–2011).

Between 2001 and 2011, 158 untagged adult Atlantic salmon were caught at Woolston weir within the River Mersey by the Environment Agency (England & Wales). A recent study, which followed 30 salmon caught at the weir, found that eight successfully ascended into the upper reaches of the River Mersey (Billington 2012). During this period, sampling effort and surveillance has been extensive and, although neither a ‘run’ of smolts to sea or a defined ‘run’ of returning adult salmon has been detected, three juveniles were sampled from the headwaters. Therefore, we argue that although the Mersey is not yet a self-sustaining population, the river is in the early stage of an on-going process of natural recolonization, following substantial improvements in overall river health. Assuming such improvements can be maintained, one can envisage that their colonization process could be actively encouraged once the source of recolonizing adults has been identified. Moreover, if recolonizing fish are shown to originate from similar (generally local) rivers, in which resident salmon are locally adapted, it seems probable that these fish may also exhibit some preadaptation to any proximal un-colonized river. Certainly, in studies of the Selja, Estonia (Vasemägi et al. 2001), and the Thames, UK (Griffiths et al. 2011), recolonization appears to be predominantly by salmon from proximal rivers in the face of massive stocking with exogenous fish. Now that a comprehensive microsatellite baseline, which includes fish from throughout their European range (Griffiths et al. 2010; SALSEA consortium, unpubl. data), is available for Atlantic salmon, such identification is finally feasible.

The objective of this study was to identify the origin of adult and juvenile salmon sampled from the River Mersey between 2001 and 2011. To do this, we genotyped a sample of 149 Mersey salmon, three of which were juveniles, with a suite of 14 microsatellite loci used previously to assemble a baseline of genetic data from populations of salmon from across the southern part of their European range (Griffiths et al. 2010). The Mersey genotypes were then assigned to a compiled baseline of probable source populations, which were taken from a previous study by Griffiths et al. (2010), and supplemented with additional populations from the SALSEA-Merge database (SALSEA consortium).

## Materials and Methods

### Fish sampling

Ascending adult salmon were caught in a fish trap fitted to a Larinier fish pass built into Woolston weir on the River Mersey, 6.2 km upstream of the tidal limit. Fish were captured during August–October in the years 2001, 2002, and 2005–2010, with fishing effort being ad hoc over this period. Salmon (all adults) were carefully removed from the trap, measured, weighed, and scales removed for aging and genotyping. The three juvenile fish sampled were caught during routine Environment Agency electric fishing surveys in the upper reaches of various Mersey tributaries. The total sample for genetic analysis was 149 Atlantic salmon (146 adults and three juveniles; Supporting Information 1).

### DNA extraction

Genomic DNA was extracted from individual scales using a Chelex protocol (Estoup et al. 1996). DNA from individual fish was genotyped using a panel of 14 apparently neutral loci: Ssa14 (McConnell et al. 1995); Ssa202, SSsp3016, Ssa197 (O'Reilly et al. 1996); SsaF43 (Sánchez et al. 1996); SSspG7, SSsp1605, SSsp2210, SSsp2201, and SSsp2216 (Paterson et al. 2004); Ssa171, Ssa289, Ssa157, and SsaD144 (King et al. 2005). The loci were amplified within three multiplexed polymerase chain reactions (PCR), comprising: (1) SSspG7, Ssa14, Ssa202, SSsp3016; (2) Ssa197, SsaF43, SSsp1605, SSsp2210, SSsp2216; (3) SsaD157, Ssa171, Ssa289, SsaD144, SSsp2201. Loci were multiplexed on the basis of size using the Beckman Coulter three dye system (Appendix 1).

PCR reactions were carried out in 10-μL reactions containing approximately 50ng of extracted Atlantic salmon template DNA, 3 μL water, 5 μL of QiagenTaq PCRMastermix, and 1μL of primer mixture (Appendix 1). PCR conditions were as follows: an initial denaturation step of 5 min at 95**°**C, followed by a touchdown PCR consisting of eight cycles with a 30-sec denaturation step at 95°C, a 90-sec annealing step starting at 62°C, and decreasing the temperature 2°C every two steps until the touchdown temperature of 47°C was reached, with 3 min of extension at 72°C. The reaction ended with a final 10-min extension at 72°C.

The size of the fluorescently labeled PCR products was determined using a Beckman Coulter CEQ8000 automatic DNA sequencer and the associated fragment analysis software (Beckman Coulter, Inc., Fullerton, California). Data were checked for scoring errors due to stutter peaks, large allele dropout, and null alleles using the program MICRO-CHECKER v2.2.3 (van Oosterhout et al. 2004).

### Analysis

**Figure 2:**
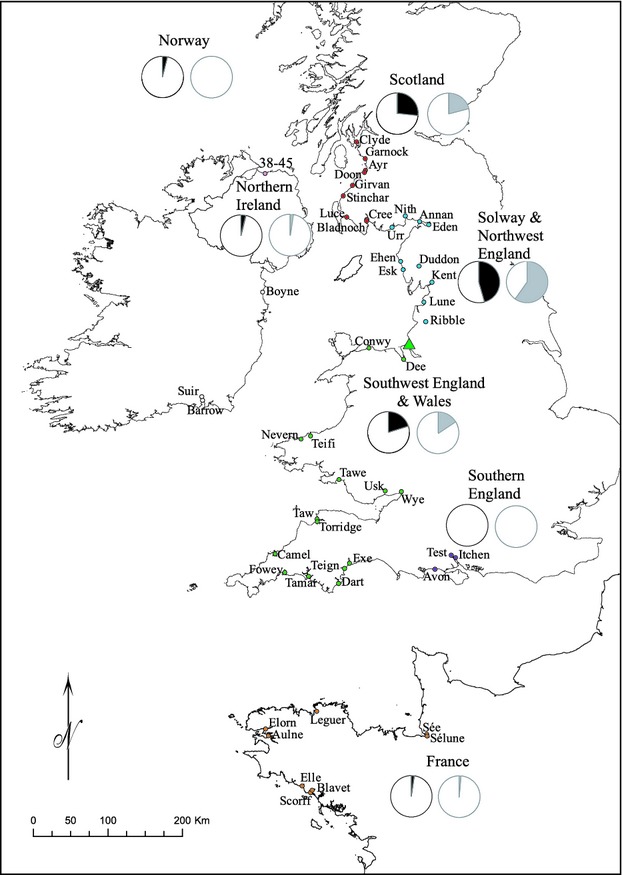
Map showing the reporting regions of origin for the 113 adults subjected to assignment analysis. Points show the mouth locations of all rivers included within the baseline, excluding those in Norway. Rivers are color coded according to their designated reporting regions: Scotland: Red; Solway & northwest England: Blue; southwest England & Wales: Green; Southern England: Purple; France: Orange; Northern Ireland: Pink (N.b. Northern Ireland rivers enter Lough Neagh and share a common estuary – 38: Upper Bann; 39: Agivey; 40: Blackwater; 41: Clogh; 42: Grillagh; 43: Kells Water; 44: Moyola; 45: Six Mile). Pie charts show the proportion of Mersey samples assigned to each reporting region in GeneClass 2 (left) and ONCOR (right).

The genetic baseline used in this study represents a subset of the database developed by Griffiths et al.(2010), supplemented with genotypes from additional populations from rivers in Ireland, eastern Scotland, and Norway from the SALSEA-Merge database (SALSEA consortium, unpubl. data) to cover potential source rivers. The baseline comprised of 5194 fish from 129 sampling sites within 60 rivers (Fig. 2; Supporting Information 2). Assignment analyses were undertaken at the level of river and to reporting regions (see below). For assignment to river, where multiple samples were available from an individual baseline river, data from all sites were pooled prior to assignment.

In order to address the possibility that adult salmon sampled in the Mersey were salmon farm escapees, four populations from Norway were included in the baseline as surrogates for farmed fish. The vast majority of fish farmed in Britain are descended from Norwegian stock (Knox and Verspoor 1991), and recent research indicates a high degree of similarity between the genetic signatures of farmed fish and those of wild Norwegian salmon (Gilbey, *pers. comm*.).

### Statistical treatment

FSTAT was used to calculate the number of alleles at each locus as well as each locus' allelic richness. Pairwise *F*_ST_ values were calculated between rivers using the program FSTAT as previous studies have shown that for populations with very low *F*_ST_ (<0.1), assignment programs can be unreliable (Latch et al. 2006). Deviations from Hardy–Weinberg equilibrium were tested for using Arlequin v3.5 (Excoffier and Lischer 2010) and critical levels of significance were adjusted using the sequential Bonferroni procedure for multiple tests (Rice 1989). To test the effectiveness of the baseline, the Leave-one out test, where each fish is systematically removed from its baseline population before having its own origins estimated using the rest of the baseline, was implemented in ONCOR (Kalinowski et al. 2008) and GeneClass 2 (Piry et al. 2004). Following these tests and the recommendations of Beacham et al. (2001), the rivers were grouped into broader, genetically based, reporting regions adapted from those proposed by Griffiths et al. (2010) for this part of the species' range.

### Defining reporting regions

Reporting regions were created by pooling data from rivers based on their genetic similarity. Genetically similar groups were identified using the programs BAPS 5 (Corander et al. 2003) and STRUCTURE v. 2.3.3 (Pritchard et al. 2000). In BAPS, this was performed by using the ‘clustering of groups of individuals’ function and setting the maximum number of groups to 10, 20, 30, 50, and 60, successively. STRUCTURE was run three times independently using the admixture ancestry model with 250,000 Markov chain Monte Carlo (MCMC) replicates after a burn-in of 50,000, assuming 1–15 populations. The process was repeated three times at different starting points along the MCMC chain. The most likely number of distinct genetic groups was inferred using the Δ*K* method of Evanno et al. (2005).The reporting regions were then also tested for effectiveness for assignment using the Leave-one out tests in ONCOR and GeneClass 2.

### Assignment

Genetic stock assignment of the Mersey salmon to the designated reporting regions was carried out using the programs ONCOR, which uses a maximum likelihood approach to assignment, and GeneClass 2, which uses a Bayesian approach. These methods have proven to be significantly more effective at assignment than previous distance-based methods (Cornuet et al. 1999). ONCOR was run under standard conditions and GeneClass 2 was run using the Rannala and Mountain (1997) algorithm.

A recognized flaw of assignment methods is the assumption that the source population is included within the baseline (Cornuet et al. 1999). In order to test this assumption, the exclusion method of assignment was performed according to Vasemägi et al. (2001).

## Results

Of the 149 Mersey salmon sampled, 134 adults and one juvenile were successfully amplified at 10 or more loci of 14; unfortunately, due to the condition of the very limited amount of scale material collected, amplification was not successful from two of the three juveniles sampled. MICRO-CHECKER found no evidence of scoring errors due to stutter peaks or allele dropout. Evidence of null alleles was found at some loci; of the 45 indications of null alleles, 10 were associated with locus SSspG7 and 8 with Ssa197. Previous work by Griffiths et al. (2010) showed the removal of loci with null alleles to be slightly detrimental toward the process of assignment. The issue has also been addressed by Carlsson (2008), who, from simulations, concluded that although null alleles can cause a slight overestimation of *F*_ST_ and a slight reduction in assignment power, their inclusion is not likely to alter the outcome of assignment; therefore, these loci were not removed from the analyses.

### Genetic diversity within the baseline

The total number of alleles per locus ranged from eight in Ssa14 to 43 in SsaD157 and SsaD144 and allelic richness ranged from 2.27 in Ssa14 to 9.45 in SsaD144 (Table 1). Heterozygosity was generally high, but ranged from 0.934 in SSsp2201 to 0.366 in Ssa14.

**Table 1:** Total number of alleles in each locus and the allelic richness per locus over all baseline populations

Locus	No. alleles	Allelic richness	Observed heterozygosity
SSspG7	26	7.61	0.834
Ssa14	8	2.27	0.366
Ssa202	24	6.98	0.855
SSsp3016	20	7.32	0.861
Ssa197	33	8.37	0.871
SsaF43	13	4.05	0.666
SSsp1605	15	5.66	0.786
SSsp2210	18	5.98	0.755
SSsp2216	21	7.52	0.883
SsaD157	42	9.04	0.924
Ssa171	37	7.52	0.871
Ssa289	12	3.69	0.632
SsaD144	43	9.45	0.930
SSsp2201	37	9.4	0.934

### F_st_s and Hardy–Weinberg

The average inter-river *F*_ST_ for all rivers included in the baseline was 0.036 (Supporting Information 3), which was less than the 0.05 recommended by Latch et al. (2006) for 97% accuracy of assignment. This was reduced to 0.0298 when looking within the UK alone, 0.027 after excluding populations from Ireland, and 0.019 after excluding the populations from Southern England. This confirmed the need to use reporting regions rather than individual rivers for subsequent assignment analysis. Twenty-seven alleles (1%) were found to be out of Hardy–Weinberg equilibrium after Bonferroni correction (Supporting Information 4). As no allele or population was found to be consistently out of HW, no data was excluded due to this test.

### Population structuring

**Figure 3:**
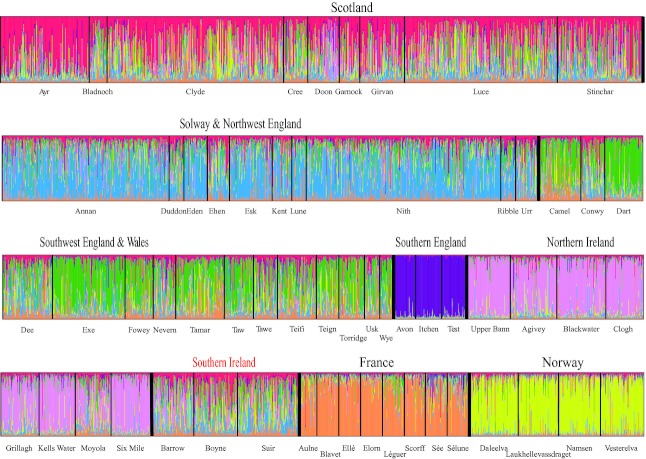
STRUCTURE plot showing estimated proportions of the coefficient of admixture of each individual's genome that originated from population *k*, for *k* = 7. Each individual is represented by a column. Thin black bars separate individual rivers, for which names are given below the graphic. Thick black bars separate reporting regions, for which the names are given above the graphic. The rivers from Southern Ireland are in red because they were removed from the assignment analysis.

The Δ*K* method identified the optimum number of genetic units from the STRUCTURE analysis to be seven (Appendix 2). The clustering of rivers within the corresponding *k* = 7 run agreed strongly with the clustering by BAPS, except for the following exception: BAPS placed the southern Irish rivers, the Barrow, Boyne, and Suir, together with the rivers from Scotland, whereas STRUCTURE identified that each river contained a mosaic of genetic signatures matching those from Northern Ireland, Scotland, and from around the Solway Firth (Fig. 3). Due to this discrepancy, which reduced the distinction between the other reporting regions, the genotypes from the Barrow, Boyne, and Suir were removed from baseline used in assignment analyses. This led to their being seven genetically based reporting regions, named as follows: Scotland, Solway & northwest England, southwest England & Wales, Southern England, Northern Ireland, France, and Norway (a surrogate for Scottish farmed fish) (Figs. 2, 3).

### Baseline test

The leave-one-out test found 46.5% in ONCOR and 47.5% in GeneClass 2 (results not shown) of fish correctly assigned back to the river from which they were sampled. After the formation of reporting regions, the proportion of correctly self-assigned individuals increased to 78% in GeneClass 2 and 79% in ONCOR (Table 2).

**Table 2:** The percentage of individuals that correctly assigned back to their own reporting region and the reporting region that contained the highest proportion of wrongly assigned individuals

Reporting Region	Correctly self-assigned		Largest incorrect assignment		
	
	GeneClass 2	ONCOR		GeneClass 2	ONCOR
Scotland	70.0%	72.0%	Solway and northwest England	10.7%	10.3%
Solway and northwest England	76.6%	76.1%	Southwest England and Wales	9.3%	9.3%
Southwest England and Wales	70.8%	71.8%	Solway and northwest England	10.6%	10.5%
Southern England	97.2%	97.1%	Solway and northwest England*	0.7%	0.7%
Northern Ireland	89.2%	89.0%	Scotland	4.1%	4.1%
France	89.1%	88.8%	Southwest England and Wales	5.5%	5.5%
Norway	90.4%	90.2%	Scotland	4.3%	4.2%

* GeneClass 2 assigned one individual from Southern England to Solway and northwest England, Southwest England and Wales, Northern Ireland and France.

**Table 3:** Results of assignment of adult Mersey fish to the seven reporting regions. Values show the exact number and percentage of individuals assigned to each reporting region in GeneClass 2 (left columns) and ONCOR (right columns)

Reporting region	GeneClass 2		ONCOR	
	
	*n*	%	*n*	%
Scotland	34	26.87	26	23.01
Solway and northwest England	49	44.03	67	59.29
Southwest England and Wales	18	18.66	15	13.27
Southern England	1	0.75	0	0.00
Northern Ireland	4	3.73	3	2.65
France	3	2.24	2	1.77
Norway	4	3.73	0	0.00

### Assignment results

Exclusion analysis found that for 21 of the 135 salmon sampled from the Mersey, the probability of their assigning to any of the recognized reporting regions was less than 0.05 (Supporting Information 5). Therefore, the results of assignment analysis for these individuals are not considered further, but can be found in Supporting Information 1.

Genetic assignment showed the remaining salmon from the Mersey to have a variety of different origins (Table 3). Both GeneClass 2 and ONCOR found the largest proportion of the Mersey salmon to be from the reporting region defined as Solway & northwest England (44% and 59%, respectively). Both methods also found the next biggest contributing regions to be Scotland, followed by Wales & southwest England. Two fish were assigned to France by ONCOR, while the same two fish and one other were assigned to France by GeneClass 2. Three fish were assigned to Northern Ireland in both GeneClass 2 and ONCOR, and one other was also assigned to Northern Ireland in GeneClass 2. Four salmon were assigned to Norway in GeneClass 2, but none were assigned to Norway in ONCOR. The single juvenile that was sufficiently well genotyped to allow meaningful assignment was assigned to Solway & northwest England by both programs (see Supporting Information 1 for likelihood scores for assignment to each reporting region).

## Discussion

This study aimed to identify the origins of Atlantic salmon recolonizing the river Mersey and in doing so, has revealed some limitations for genetic assignment of wild salmon within this region. Although most of the salmon now entering the Mersey could not be assigned to an exact river of origin, by identifying distinct genetic signatures of groups of salmon rivers, we were able to identify their region of origin with a high degree of probability. The reporting regions identified here matched those identified by Griffiths et al. (2010) and, according to the results of the self-assignment tests, they appear to be valid units for assignment. For some reason, possibly unidentified salmon translocations, the Southern Irish rivers used in this study contained genotypes that failed to stand alone as a distinct reporting region. For this reason, these rivers were removed from the assignment analysis.

The genetic baseline used for assignment of Mersey fish was a subset of the populations used in the ASAP (Griffiths et al. 2010) and SALSEA (SALSEA consortium, unpublished) projects. Such a baseline was anticipated to provide comprehensive coverage of potential rivers of origin for those salmon now entering the Mersey. Nonetheless, even with such detailed coverage, the possibility remained that some fish might not assign to a population or region within the baseline. Accordingly, to address this possibility, we undertook exclusion analysis. This analysis found that 21 of the 135 salmon characterized did not assign to any of the reporting regions in our baseline; this may be because these fish really do originate from a population outside the area covered by our baseline, or may indicate that their genetic signatures are too general to assign to any reporting region with a sufficiently high score (above 0.05). This left 113 adults and one juvenile for assignment analysis, which identified multiple origins for salmon currently entering the river Mersey (Table 3). This finding is not unusual as previous studies also show recolonization from multiple source rivers (e.g., the River Seine, Perrier et al. 2010). Indeed, this should be beneficial for the long-term survival of any newly established population, as the potentially increased genetic variability should provide a broader basis for adaptation to local and possibly changing conditions.

The Mersey is found to be on the border between two of the reporting regions designated in this study. The majority of salmon in the Mersey clearly originate from rivers north of this border and, in particular, the Solway & northwest England reporting region. Although this finding is not on its own surprising (the southernmost river of this reporting region being the Ribble, the mouth of which is approximately 40 km north of the Mersey), it was striking that so few (15/113 ONCOR; 18/113 GeneClass 2) appeared to have origins in the neighboring southwest England & Wales region (a trend reflected in assignment to river; Supporting Information 1). In particular, this reporting region contains the river Dee, a highly productive salmon river that enters the sea in close proximity to the Mersey; the estuaries of these two major rivers are separated by the 11-km-wide Wirral peninsula. This finding may be due to the prevailing clockwise gyre in the eastern Irish Sea and an associated current, which for much of the year runs southwards down the northwest coast of England (Heaps and Jones 1977). Presumably, it is this current, which carries some homing adult salmon past their natal rivers and southwards toward the Mersey, while simultaneously acting to move fish from the rivers of north Wales away from the Mersey.

This study finds conclusive evidence that, despite their well-known homing capabilities (Stabell 1984), Atlantic salmon can stray into distant rivers. Two or three fish (depending on method) were assigned to France by the programs. Previous work has shown that long distance colonization does occur; for example, a study of recolonizers in the Séine (Perrier et al. 2010) showed two of seven fish assigned to a foreign baseline group better than any of the five French regions included in their analysis. A study by Griffiths et al. (2011), which found one of 16 salmon sampled from the Thames to be from a French population, again demonstrates that salmon may stray relatively long distances to rivers in England.

An important caveat is that, despite evidence confirming that some of the stray adults caught in the weir do ascend into the Mersey's upper reaches (Environment Agency 2012), within the limits of this study we cannot determine which of the 135 genotyped adults would have ascended the river further and which would have left the catchment. However, the one juvenile, for which there was enough material to amplify the DNA reliably, assigned to the Solway & northwest England region. We refrain from making major conclusions based on a single individual; however, the importance of this juvenile should not be overlooked. This result suggests that not only are rivers in the Solway and northwest England region the biggest source of strays, but also (because of their larger numerical contribution and their preadaptation to similar in-river conditions in their proximal rivers of origin) that salmon from this region are the most likely to successfully reproduce in the river Mersey at this time.

### Farmed salmon

Four salmon populations from Norway were included in the baseline to represent the genetic signature of farmed fish of Norwegian-origin, which we considered might be a possible source of adult fish entering the Mersey. However, the results for this component of the analysis were inconclusive; four of the 113 Mersey adults assigned to Norway with GeneClass 2, whereas none were assigned to Norway with ONCOR. This discrepancy may indicate that the actual source population of these fish is not present within the baseline, as previous studies have concluded (e.g., Perrier et al. 2010). However, additional evidence indicating a Norwegian genetic signature in the possible sources of Mersey fish comes from the STRUCTURE analysis (Fig. 3). Some Scottish rivers such as the Clyde and Luce show clear evidence of resident salmon parr with Norwegian genetic signatures. These ‘Norwegian’ fish may be descendants of fish farm escapees, but it is also possible that this reflects a shared common ancestry of northern salmon populations. Whatever their origins, one of our methodologies indicates that fish with at least a partial Norwegian signature are entering the Mersey. At this time, however, discrepancies in our assignment prevent us from making a firm conclusion, but improving the baseline with the addition of known hatchery stock may help to resolve this issue.

### Difficulty of assignment

To date, no study has made use of such an extensive baseline for identifying the origin of unknown Atlantic salmon. Although the epitome of genetic stock identification applications would be to identify any salmon to its river or possibly tributary of origin, for this part of the species' range (western Britain/eastern Ireland) at least, this is beyond current means. The results of the leave-one-out test showed that less than one in two fish could be correctly assigned back to their river of origin; unfortunately, such a figure is insufficient for meaningful assignment. This was somewhat to be expected as previous research by Griffiths et al. (2010), also found lower accuracy of assignment in this region (Ireland, the west coast of Scotland, northwest England, and Wales), compared with that obtained when assigning to more southerly salmon populations. Inter-river *F*_ST_ values of 0.02 within each of the designated UK reporting regions analyzed in this study, and many pairwise inter-river *F*_ST_ values of less than 0.01 underline the inability to assign to individual rivers within this area; these values are far below the 0.05 suggested for 97% assignment accuracy (Latch et al.^2006^).

Key to improving the accuracy of genetic assignment is improving the genetic distinction between populations within a baseline. One way of doing this might be to increase the number of markers used, either via the addition of more microsatellites, or with the use of Single Nucleotide Polymorphisms (SNPs) (Beacham et al. 2011). Currently, however, the utility of SNPs for assignment purposes remains a topic of considerable discussion (e.g., Morin et al. 2004; Beacham et al. 2011; Hauser et al. 2011). Another key approach is to reduce the sampling error, that is, the difference between the estimated allele frequencies and the allele frequencies in the actual population (Beacham et al. 2011). This could be achieved by increasing the sampling size of the baseline populations, which, although not ideal, might be less effort than the cross-calibration required with the addition of extra microsatellite loci (e.g., Ellis et al. 2011).

### River restoration as a fisheries management tool

Overall, this study and others like it (e.g., Knutsen et al. 2001; Schreiber and Diefenbach 2005; Anderson and Quinn 2007; Kiffney et al. 2009; Perrier et al. 2010; Griffiths et al. 2011) serve to underline the value of river restoration as an effective alternative to stocking to promote the recolonization of rivers from which salmonids have been previously extirpated. Additionally, such an approach is likely to yield broader ecological benefits for a river ecosystem as a whole. For example, improvements in water quality have been shown to promote increased biodiversity of riverine invertebrate fauna (Chadwick et al. 1986) and the return of larger animals, for example otters, in part due to improved water quality and partly due to increased availability of fish as food (Pountney et al. 2009; Crawford 2010).

Alternatively, in situations where the need for fish population restoration is urgent – for example, post-pollution mitigation – then assignment studies such as this offer, in combination with river restoration, robust insights as to which populations might best serve as donors for translocation, and thus more rapid recolonization.

## Conclusion

In conclusion, this study overcomes limitations in genetic assignment in order to ascertain the origins of Atlantic salmon recolonizing the river Mersey. Fish entering the Mersey appear to be from multiple regions primarily within England, Scotland, and Wales, and, in particular, from the rivers in close proximity to the Solway Firth and the northwest of England. This key finding highlights an apparent clockwise direction of straying by Atlantic salmon in this region, which we speculate to be due to the clockwise Gyre in the eastern Irish Sea. The one successfully analyzed juvenile assigned consistently to this same region, which may indicate that not only is this region responsible for the greatest number of strays, but that these strays are also the most likely to successfully reproduce in this river. This study also finds that a small fraction of their colonizers are from northern Ireland, whereas a similarly small proportion appears to originate from France. The evidence suggests that salmon farm escapees with a distinct Norwegian signature may be a fraction of the recolonizers; however, incongruence between the methods used prevented firm conclusions on this topic. Although the information gained from this study increases our scientific understanding of the salmon life cycle, our findings are also especially useful for river management, as they demonstrate clearly the benefits of river restoration as a *bona fide* methodology for the re-establishment of salmonid populations in rivers from which they have been previously extirpated; our results also serve to reconfirm the capacity for straying in this species otherwise famous for its homing ability.

## Figures and Tables

**Figure A1:**
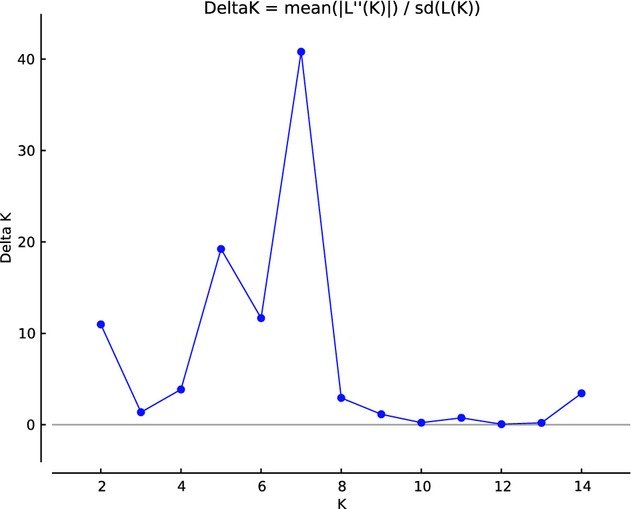
Δ*K* plot to identify the optimum number of genetic units within the data in the program STRUCTURE

## Data archiving statement

Data for this study are available at: http://datadryad.org/ DOI: 10.5061/dryad.ck461.

## Appendix

**Table A1:** Dyes, concentrations, and multiplexes for microsatellite primers.

## Supporting Information

**Table S1.** Mersey salmon origins and assignment details.

**Table S2.** Sample sites and rivers used in the genetic baseline.

**Table S3.** Pairwise Fst values between baseline rivers (calculated in Fstat).

**Table S4.** Assessment of Hardy–Weinberg equilibrium at each locus for each baseline river (calculated in Arlequin).

**Table S5.** Exclusion analysis performed using the Cornuet et al. (1999) algorithm.
